# O‑Island 28 encodes a type I secretion and RTX adhesion system regulated by RstA and required for early EHEC O157:H7 adherence

**DOI:** 10.1080/19490976.2025.2609461

**Published:** 2025-12-27

**Authors:** Tianshui Niu, Mengqian Huang, Fei He, Xianjuan Yu, Aifeng Chen, Yan Shi, Jiaying Han, Xiang Lian, Junwei Su, Yutao Liu, Chuhui Ru

**Affiliations:** a Department of Pulmonary and Critical Care Medicine Center, Zhejiang Hospital of Integrated Traditional Chinese and Western Medicine/Hang Zhou Red Cross Hospital, Hangzhou, People's Republic of China; b School of Life Sciences, Tianjin University, Tianjin, People's Republic of China; c Department of Infectious Diseases, The Affiliated Xiangshan Hospital of Wenzhou Medical University; Xiangshan First People’s Hospital Medical and Health Group; Ningbo Fourth Hospital, Ningbo, People's Republic of China; d State Key Laboratory for Diagnosis and Treatment of Infectious Diseases, National Clinical Research Center for Infectious Diseases, Collaborative Innovation Center for Diagnosis and Treatment of Infectious Diseases, The First Affiliated Hospital, Zhejiang University School of Medicine, Hangzhou, People's Republic of China

**Keywords:** Enterohemorrhagic *Escherichia coli*, O-Island 28, type I secretion system, two-component system, virulence regulation

## Abstract

Enterohemorrhagic *Escherichia coli* (EHEC) is a leading foodborne pathogen worldwide that causes severe diarrheal disease, hemorrhagic colitis, and hemolytic uremic syndrome, representing a significant threat to public health. O-islands are discrete genomic regions absent from nonpathogenic *E. coli* K-12 but present in EHEC O157:H7, many of which correspond to or overlap with pathogenicity islands (PAIs) that contribute to virulence. Among these O-islands, O‑Island 28 (OI‑28) in EHEC O157:H7 is a conserved genomic island predicted to encode a complete type I secretion system (T1SS) and two RTX family proteins, but its role in pathogenesis has remained unclear. Here, we show that deletion of OI‑28 markedly reduces epithelial adherence and intestinal colonization in mice without affecting in vitro growth. Mechanistically, OI‑28 is activated by the response regulator RstA, and RstA binds directly to the OI‑28 regulatory region. Consistent with the calcium-dependent folding of RTX adhesins, extracellular Ca^2+^ enhances OI‑28 expression and T1SS-dependent adherence in an RstA-dependent manner, and dietary calcium depletion reduces early colonization in vivo. Comparative genomics further demonstrated that OI‑28 is required for the colonization of multiple pathogenic *E. coli* strains. Collectively, these findings demonstrate that OI‑28 is an RstA‑activated, calcium‑responsive T1SS secretion system that is conserved across pathogenic *E. coli* strains and is essential for efficient epithelial adherence and early intestinal colonization.

## Introduction

Enterohemorrhagic *Escherichia coli* (EHEC) is one of the major foodborne pathogens responsible for significant public health concerns worldwide.^1^ EHEC O157:H7 infections are commonly associated with the consumption of contaminated food or water and can lead to illnesses ranging from diarrhea to severe hemorrhagic colitis (HC) and hemolytic uremic syndrome (HUS).^2^ EHEC O157:H7 is the serotype most frequently implicated in large outbreaks and is distinguished by its extremely low infectious dose and potential for life-threatening complications.^3^^,^^4^

The pathogenesis of EHEC O157:H7 involves several key virulence factors, including the production of Shiga toxins (Stx1 and Stx2) and the formation of attaching and effacing (A/E) lesions on intestinal epithelial cells.^5^ The locus of enterocyte effacement (LEE) mediates A/E lesion formation, enabling intimate adherence to the intestinal epithelium and driving microvilli disruption and pedestal formation.^6^ The LEE‑encoded type III secretion system (T3SS) functions as a molecular syringe that secretes effectors into host epithelial cells.^7^ In addition, Shiga toxins are encoded by lysogenic phage genes and belong to the AB_5_ family, which consists of one enzymatic A subunit bound to a pentameric B subunit.^8^ More importantly, additional adhesins, outer membrane proteins, and secreted toxins further promote colonization and persistence during infection.^9^

The type I secretion system (T1SS) is a well‑known one‑step secretion pathway in Gram‑negative bacteria.^10^ It comprises of an inner‑membrane ATP‑binding cassette (ABC) transporter, a membrane fusion protein (MFP), and an outer‑membrane protein (OMP), which together form a continuous channel that spans the bacterial envelope, enabling direct secretion of substrates from the cytoplasm to the extracellular environment. The T1SS is responsible for exporting a wide array of effector proteins, including Repeats‑in‑Toxin(RTX) proteins, enzymes, and adhesins. Representative secreted substrates include the hemolysin HlyA (~110 kDa), the heme carrier HasA, and large adhesins such as LapA (~900 kDa).^11-13^ These secreted proteins often play key roles in bacterial virulence and host colonization.

Among the major substrates of the T1SS are a family of proteins known as RTX toxins, which are defined by characteristic glycine‑ and aspartate‑rich repeat motifs that coordinate calcium (Ca²⁺) binding.^14^ In the Ca²⁺‑limited cytoplasmic environment, RTX proteins remain largely disordered; however, upon secretion through the T1SS into the Ca²⁺‑rich extracellular milieu, they fold rapidly into stable and active conformations, enabling their diverse cytotoxic or adhesive functions.^15^^,^^16^ In pathogenic bacteria, T1SS-secreted repeats-in-toxin (RTX) proteins are critical for pathogenesis and mediate the initial interactions between pathogens and the host.^17^ For example, the SPI‑4‑encoded adhesin SiiE in *Salmonella* enterica contributes to intimate epithelial adherence,^18^ the hemolysin HlyA in uropathogenic *E. coli* promotes host cell membrane disruption and bacterial entry,^19^ and the giant adhesin LapA in *Pseudomonas fluorescens* mediates attachment to biotic and abiotic surfaces, facilitating biofilm formation.^13^ Recent studies have revealed that a previously uncharacterized genomic region in EHEC O157:H7, known as O-Island 28 (OI-28), is predicted to encode a complete T1SS and two RTX toxins. However, the role of OI-28 in pathogenesis remains unclear.^20^

During infection, EHEC O157:H7 is capable of sensing changes in the intestinal environment and regulating the expression of virulence genes, which are critical for successful bacterial colonization and pathogenesis.^21^ Two‑component systems (TCSs) enable this adaptation by coupling a sensor histidine kinase (HK) to a response regulator (RR).^22^ For example, EHEC O157:H7 senses microbiota-produced riboflavin to directly activate the expression of LEE genes encoding the T3SS in the colon.^23^ In addition, other TCSs, such as PhoPQ, QseBC, and CpxRA, detect signals such as low magnesium concentrations, host-derived catecholamines, and envelope stressors, respectively.^24-26^ In particular, the RstAB TCS in EHEC O157:H7 plays a major role in the regulation of virulence, acid tolerance, and biofilm formation.^27^ RstAB in *Pseudomonas aeruginosa PAO1* is responsible for sensing high levels of external Ca^2+^ and modulating Ca^2+^ homeostasis, surface-associated motility, and the production of the virulence factor pyocyanin.^28^ Thus, the ability of EHEC O157:H7 to sense diverse signals from both the host and the gut microbiota creates a dynamic network for virulence regulation, which promotes bacterial infection.^29^

In this study, we identified OI-28 as a conserved genomic island in EHEC O157:H7 that encodes a complete T1SS (ABC transporter, membrane fusion protein, and outer membrane protein) and two RTX proteins. We showed that the deletion of OI-28 markedly reduces epithelial adherence and intestinal colonization. Domain-focused complementation demonstrated that Ig-like repeats within RTX toxins are required for adhesion and virulence-associated attachment. Mechanistically, we revealed that the response regulator RstA directly binds the OI-28 regulatory region and activates its expression. Finally, we show that the T1SS in OI-28 is conserved and broadly associated with the colonization capacity of pathogenic *E. coli*. Together, these results reveal a T3SS-independent adhesion pathway, which may act as a promising anti-virulence target.

## Results

### An evolutionarily conserved OI‑28 encodes a predicted T1SS and RTX toxin

**Figure 1.**
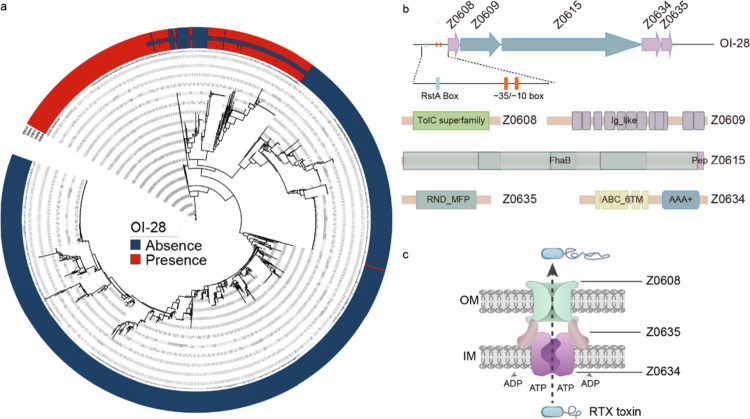
Distribution and domain organization of OI‑28. (a)Conservation of OI‑28 across 2,122 representative *E. coli* strains. The rings indicate, from outer to inner, *z0608*(*tolC28*), *z0609*(*rtxA28*), *z0615*(*rtxB28*), *z0634*(*hlyB28*), and *z0635*(*hlyD28*). Presence (red) and absence (blue) are shown. (b) Domain architecture and prediction of the putative σ⁷⁰ promoter upstream of OI‑28 using NCBI Conserved Domain Database and BPROM. The predicted −35 and −10 elements are shown together with the mapped RstA box located in the upstream regulatory region of OI-28. (c) Schematic of the OI‑28‑encoded T1SS comprising the ABC transporter (Z0634), MFP (Z0635), OMP (Z0608), and RTX toxins(Z0609, Z0615). IM, inner membrane; OM, outer membrane.

To analyze the distribution of OI-28 in *E. coli*, a total of 2,122 *E. coli* genomes were analyzed. Among these, OI‑28 is highly conserved in EHEC O157:H7 (222/222, 100%), O145:H28 (18/18, 100%) and EPEC O55:H7 (9/9, 100%). In contrast, the detection rate of OI‑28 was lower in other pathotypes, being present in 11 of 36 UPEC genomes (30.5%) and 9 of 140 other STEC genomes (6.4%), but completely absent in ExPEC (0/55, 0%) and EAEC (0/10, 0%) (Figure 1a, Supplementary Table 3). This finding indicates that the acquisition of OI-28 may confer selective advantages to highly pathogenic of EHEC O157:H7.

Detailed domain structure of the proteins in OI-28 was analyzed by NCBI Conserved Domain Database (CDD). The results showed that OI-28 in the EHEC O157:H7 strain EDL933 contains a gene cluster encoding all essential components of a canonical T1SS: an ATP-binding cassette (ABC) transporter (Z0634), a membrane fusion protein (MFP) (Z0635), and an outer membrane protein (OMP) (Z0608) (Figure 1b). Mechanistically, the ABC transporter, which is located in the inner membrane of bacteria, harnesses the energy from ATP hydrolysis to start the secretion process. This transporter usually operates as a homodimer and specifically binds to the noncleaved C-terminal secretion signal of secreted proteins (Figure 1c). The MFP acts as a structural and functional bridge between the inner and outer membranes. By spanning the periplasmic space, it connects the ABC transporter and outer membrane proteins (such as TolC). This arrangement forms a channel, providing efficient passage of secreted proteins and preventing substrate accumulation in the periplasm (Figure 1c). The OMP, located in the outer membrane, completes the channel and allows secreted proteins to enter the extracellular space^30^^,^^31^ (Figure 1c). Subcellular localization of the proteins encoded by Z0634, Z0635, and Z0608 was further predicted using PSORTb v3.0. The results showed that Z0634 is localized to the cytoplasmic membrane with a localization score of 10.0, showing internal transmembrane helices and matching the “Toxin RTX‑III translocation ATP‑binding protein”. Similarly, Z0635 was also predicted to be a cytoplasmic membrane protein (localization score 9.82), consistent with its homology to the “Hemolysin secretion protein D”, which functions as a membrane fusion component of the type I secretion system (T1SS). Z0608 was predicted to localize to the outer membrane (localization score 10.0) and matched an “outer membrane integral membrane protein” (accession ID 11354392).

Together, these results indicate that Z0634, Z0635, and Z0608 correspond to the three core structural elements of a T1SS, supporting that O‑Island 28 encodes a complete functional T1SS apparatus.

In addition, OI-28 contains two genes predicted to encode two large RTX family proteins, characterized by the canonical RTX domain with glycine- and aspartate-rich repeats (Figure 1b). These glycine- and aspartate- rich repeats domains have been associated to virulence.^32^ Furthermore, the RTX family of proteins contains a well-conserved immunoglobulin-like (Ig-like) domain at the C-terminus of the protein, a structural motif often implicated in mediating protein-protein interactions and bacterial adhesion^33^ (Figure 1c). Subcellular localization analysis predicted that the RTX family proteins encoded within OI‑28 are likely associated with both the outer membrane and the extracellular environment, suggesting that they are surface‑exposed or secreted factors that may play a crucial role in host cell attachment and early colonization. Based on their sequence similarity and functional analyzes, the five genes in OI‑28 were named as *hlyB28*(*z0634*), *hlyD28*(*z0635*), *tolC28*(*z0608*), *rtxA28*(*z0609*), and *rtxB28*(*z0615*) encoding the ATP‑binding transporter Z0634, membrane fusion protein Z0635, outer membrane protein Z0608, and two large RTX adhesins Z0609 and Z0615, respectively.

Collectively, our data suggests that OI-28 encodes a functional T1SS/RTX system with evolutionary conservation among pathogenic *E. coli*, supporting its potential relevance in pathogenesis.

### OI‑28 supports early adherence and colonization

**Figure 2.**
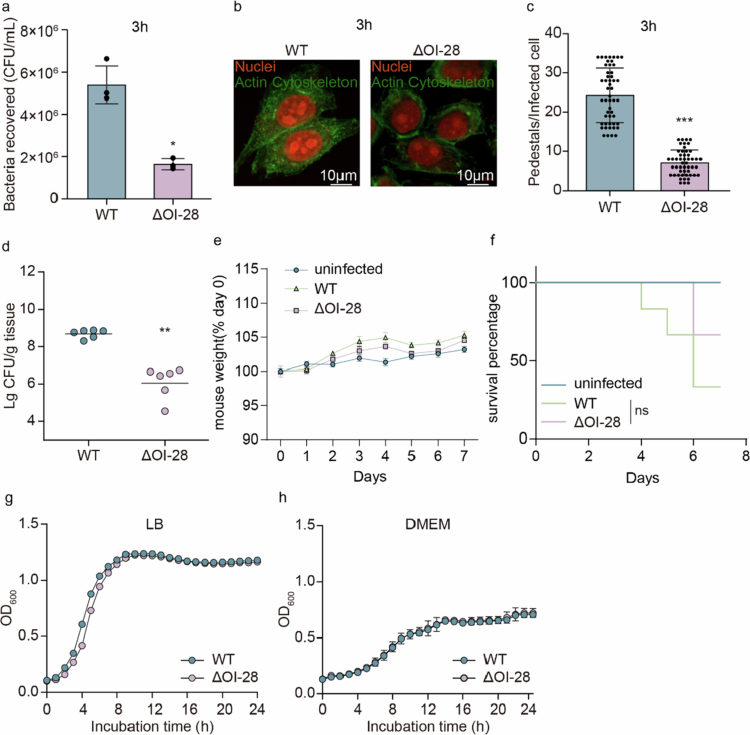
OI‑28 supports early adhesion and colonization. (a) Adherence of WT and ΔOI‑28 strains to Caco-2 cells at 3 h p.i. (*n* = 3). (b) Representative FAS images; nuclei (propidium iodide, red), actin (FITC‑phalloidin, green). Arrowheads indicate pedestals. Scale bar, 10 μm. (c) Quantification of pedestals per infected cell, *n* = 50 cells per strain. (d) Comparison of intestinal colonization by the WT and ΔO1-28 strains in the mouse distal colon. Data points represent individual mice; horizontal bars denote medians (*n* = 6). (e) Body weights of mice infected with WT or ΔOI‑28, as well as uninfected controls, were monitored daily for 7 days. Data are presented as the percentage of the initial body weight on the day of infection (day 0); curves represent mean values (*n* = 6). (f) Survival curves of mice infected with WT or ΔOI‑28 strains, as well as uninfected controls (*n* = 6). (g, h) Growth curves in LB (e) and DMEM (f) (*n* = 3). Statistics: two‑tailed unpaired Student’s t‑test (a, c, e, f) and two‑sided Mann–Whitney U test (d). *P < 0.05, **P < 0.01, ***P < 0.001; n.s., not significant.

To evaluate the contribution of OI‑28 to EHEC O157:H7 virulence, an OI‑28 deletion mutant (ΔOI‑28) of the EHEC O157:H7 strain EDL933 was constructed and verified by whole‑genome sequencing and PCR to ensure the absence of random or off‑target mutations (Fig. S1a). First, the ability of the ΔOI-28 and EHEC O157:H7 EDL933 wild-type (WT) strains to adhere to epithelial cells was analyzed by infecting Caco-2 cells at 3 hours post-infection (p.i.). At 3 h p.i., the ΔOI-28 strain exhibited a reduced adherence capacity of approximately 3.28-fold compared with that of the WT strain, highlighting the role of OI-28 in bacterial adherence at early stages of infection (Figure 2a). Following the adherence assays, the ability of ΔOI-28 to influence A/E lesions was examined via a fluorescent actin (FAS) staining assay. The FAS results revealed that, compared with the WT strain, the ΔOI-28 strain induced fewer pedestals, indicating a defect in A/E lesion formation in the ΔOI-28 strain (Figure 2b). The quantitative analysis of the FAS assay results further confirmed that the number of pedestals per cell was significantly reduced in the ΔOI-28-infected cells (Figure 2c).

To determine whether this adhesion defect persisted during prolonged infection, additional adhesion assays were performed at 6 h and 9 h p.i. The ΔOI‑28 strain showed significantly reduced attachment at 3 h, but this difference gradually diminished at 6 h and was no longer apparent at 9 h (Fig. S1b, c). Similarly, pedestal formation by the ΔOI‑28 strain was markedly reduced at the early stage of infection(3 h p.i.) and remained lower than that of WT at 6 h p.i., but this difference was no longer exist at 9 h, further supporting a role for OI‑28 in the early phase of adherence(Fig. S1d-g). These findings suggest that OI‑28 primarily contributes to the initial adherence of EHEC to host epithelial cells, facilitating efficient early colonization. In addition, we examined whether deletion of OI‑28 influences expression of the Type III secretion system (T3SS). qRT‑PCR of major T3SS structural and effector genes (*ler*, *tir*, *escN*, *espB*, *eae* and *escC*) showed no significant changes in expression compared with the WT(Fig. S1h). These results confirm that the phenotypic alterations observed in the ΔOI‑28 are specifically attributable to deletion of the OI‑28 locus, not to influence the T3SS activity.

Furthermore, in vivo colonization experiments were performed in a mouse model. The mice were orally inoculated with 1 × 10^9^ CFU of WT or ΔOI-28. The colonization assay results revealed that the bacterial colonization ability of the ΔOI-28 strain was markedly decreased compared with that of the WT strain, indicating that OI-28 is required for efficient intestinal colonization in vivo (Figure 2d). We further assessed mouse survival and body weight following infection. The results showed that ΔOI-28 did not significantly affect body weight changes and mouse mortality, suggesting that OI‑28 contributes to the intestinal colonization but not disease severity during infection (Figure 2e,f). In addition, bacterial growth curves in both LB and DMEM did not significantly differ between the ΔOI-28 strain and the WT strain, indicating that the deletion of OI-28 does not influence bacterial viability (Figure 2g,h).

Taken together, these results demonstrate that OI-28 can influence EHEC O157:H7 adhesion, actin pedestal formation, and colonization, suggesting the importance of OI-28 in EHEC O157:H7 pathogenicity.

### The T1SS and RTX proteins encoded by OI‑28 are required for adhesion and colonization.

**Figure 3.**
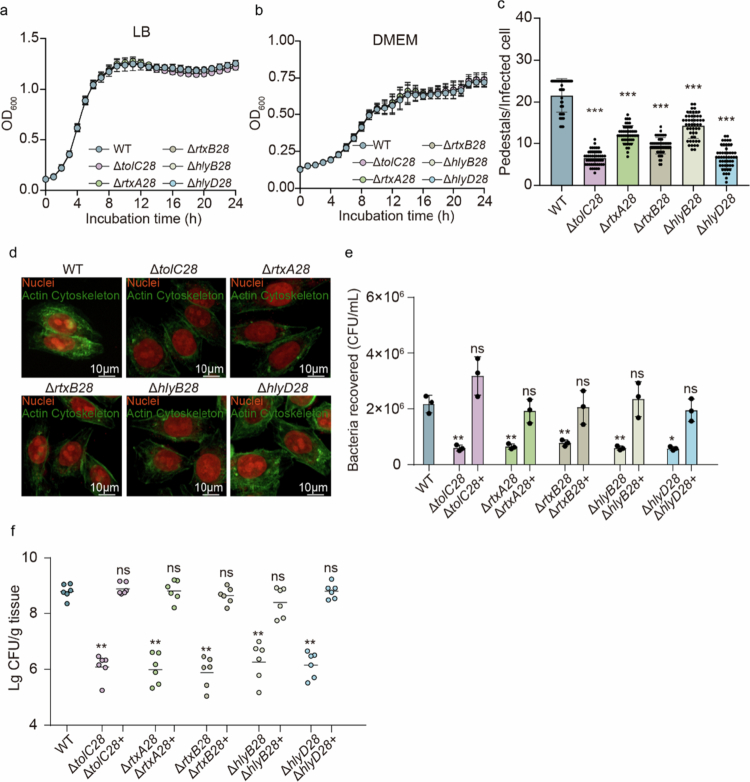
The T1SS and RTX proteins encoded by OI‑28 are required for adhesion and colonization. (a) Growth curves of the WT, Δ*tolC28*, Δ*rtxA28*, Δ*rtxB28*, Δ*hlyB28*, and Δ*hlyD28* mutant strains cultured in LB (a) and DMEM (b) for 24 h (*n* = 3). (c) FAS assay of Caco-2 cells infected with WT or mutant strains, where the nuclei and bacteria were stained with propidium iodide (red), and the actin cytoskeleton was stained with FITC-phalloidin (green). Pedestal structures appear as green puncta associated with bacterial cell arrowheads. Scale bar, 10 μm. (d) Quantification of pedestals per infected cell, *n* = 50 cells per strain. (e) Adherence capacity of the O157 WT, Δ*tolC28*, Δ*rtxA28*, Δ*rtxB28*, Δ*hlyB28*, and Δ*hlyD28* and complemented strains to Caco-2 cells at 3 h p.i. (*n* = 3). (f) Comparison of intestinal colonization by WT, mutant strains and complemented strains in the mouse distal colon (*n* = 6). Data points represent individual mice; horizontal bars denote medians. Two-tailed unpaired Student's t tests (c, e) or two-sided Mann‒Whitney U tests (f) were used to calculate *P* values. *P < 0.05, **P < 0.01, ***P < 0.001, n.s., not significant.

To further analyze the contribution of each gene in OI-28 to EHEC O157:H7 pathogenicity, single-gene deletion mutants were constructed (Δ*tolC28*, Δ*rtxA28*, Δ*rtxB28*, Δ*hlyB28*, *and* Δ*hlyD28*). Growth assays demonstrated that none of these mutants impacted bacterial growth in either LB or DMEM, which is consistent with previous observations in ΔOI-28 (Figure 3a,b).

To further evaluate the effects of these mutants on the virulence of EHEC O157:H7, cell adherence assays were performed in Caco-2 cells at 3 h p.i. The results revealed that all five mutants presented significantly lower adherence abilities than the WT strain did (Figure 3e). This observation suggests that the integrity of each gene in OI-28 can influence its attachment to host cells. Furthermore, FAS assays revealed that these single-gene mutants were markedly deficient in inducing pedestal formation on the surface of infected epithelial cells (Figure 3c). Moreover, all the mutants presented a substantial reduction in both the proportion of infected cells manifesting actin pedestals and the number of pedestals formed per cell (Figure 3d). These results indicate that all five genes within the locus are essential for EHEC O157:H7 adherence. In addition, after oral administration of each single-gene mutant, the number of bacteria recovered from the colon was significantly lower than that in mice infected with the WT (Figure 3f). This reduction in colonization capacity indicates the indispensability of each gene in OI-28 for efficient intestinal colonization.

To further verify that the observed phenotypes were specifically due to the loss of the OI‑28‑encoded T1SS, genetic complementation analyzes were performed. First, *hlyB28*, *hlyD28*, and *tolC28* were individually reintroduced into their corresponding deletion mutants. The results showed that it is sufficient to restore both in vitro adherence and in vivo colonization abilities to WT levels, confirming the necessity of these genes for OI-28-mediated virulence (Figure 3e,f). However, complementation of the RTX proteins encoded by *rtxA28* and *rtxB28* proved technically challenging, as the full-length genes could not be successfully introduced due to their considerable gene size. Domain structure analysis revealed that RtxA28 and RtxB28 contain multiple immunoglobulin-like (Ig-like) domains. Therefore, engineered genes encoding truncated versions with only two Ig-like domain copies were generated and used for complementation. Partial complementation can partially restore adherence and colonization abilities, suggesting that the complement of the Ig-like domain is required for virulence activity (Figure 3e,f).

These results suggest that all five genes within OI‑28 are required not only for efficient bacterial adherence but also for successful colonization in vivo. Among them, the RTX proteins RtxA28 and RtxB28 appear to function as adhesins mediating bacterial attachment to host cells.

### OI-28 in EHEC O157:H7 is directly regulated by RstA

**Figure 4.**
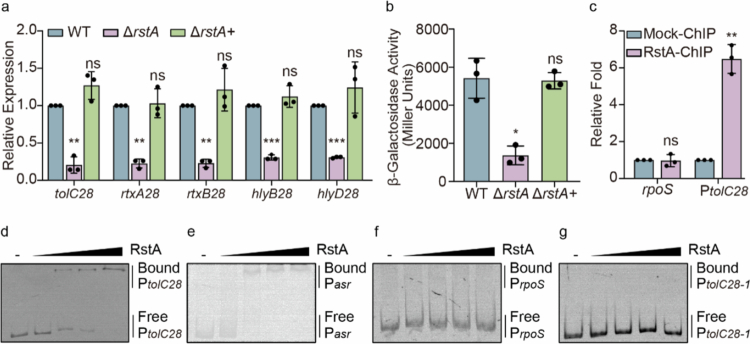
RstA directly activates OI‑28. (a) qRT‒PCR analysis of changes in *tolC28*, *rtxA28*, *rtxB28*, *hlyB28*, and *hlyD28* expression in the WT, Δ*rstA* mutant, and Δ*rstA* complementation strains (*n* = 3). (b) Transcriptional activity (*β*-galactosidase units) of the regulatory region and promoter region of *tolC28* in *lacZ* fusion constructs within the WT, Δ*rstA* mutant, and Δ*rstA* complementation strains (*n* = 3). (c) qPCR measurement of regulatory region and promoter region of *tolC28*(P_*tolC*_) enrichment in RstA ChIP samples. *rpoS* served as a negative control (*n* = 3). (d-g) EMSA results showing the specific binding of RstA to regulatory region and promoter region of *tolC28*(P_*tolC*_) (d), P*asr* (positive control) (e), *rpoS* (negative control) (f), and regulatory region and promoter region of *tolC28-*1(P_*tolC-1*_) (g). Two-tailed unpaired Student's t test (a, b, c) was used to calculate *P* values. *P < 0.05, **P < 0.01, ***P < 0.001, n.s., not significant.

Our previous study revealed that the expression of all genes within the OI‑28 were 1.47-5.26-fold downregulated in the Δ*rstA* mutant compared with the WT.^26^ Therefore, we hypothesized that RstA may regulate the expression of OI-28. To determine whether OI-28 is regulated by RstA, we quantified OI-28 expression in the WT, Δ*rstA*, and Δ*rstA* + strains via qRT-PCR. The results revealed that, compared with the WT strain, the Δ*rstA* strain presented a significant reduction in the expression of T1SS components (*hlyB28*, *hlyD28*, and *tolC28*) and RTX toxin genes (*rtxA28* and *rtxB28*) (3.25-4.85-fold). Moreover, complementation of *rstA*(Δ*rstA*+) restored the expression to WT levels (Figure 4a). The regulatory region of *tolC28* was subsequently cloned and inserted into the plasmid pHRP309, which contains a regulatory region and promoter-less *lacZ* reporter gene. The recombinant plasmid was then transferred into the WT, Δ*rstA* and Δ*rstA +* strains, which were analyzed via LacZ fusion assays. The results revealed that the activity of *tolC28* was much greater in the WT and Δ*rstA +* strains than in the Δ*rstA strain*, suggesting that *tolC28* was positively regulated by RstA (Figure 4b). These findings indicate that RstA positively affects the expression of OI-28.

**Figure 5.**
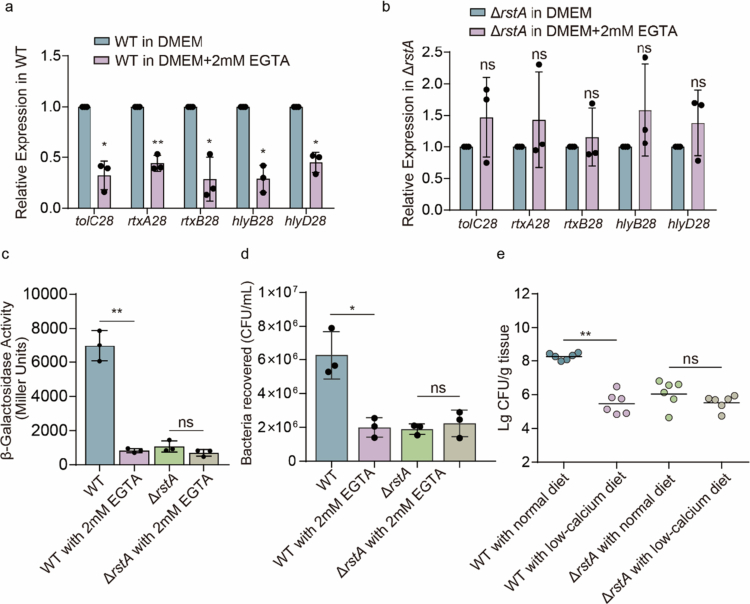
Extracellular Ca^2+^ promotes OI‑28 expression and bacterial adherence via RstA. (a, b) qRT‒PCR analysis of changes in *tolC28*, *rtxA28*, *rtxB28*, *hlyB28*, and *hlyD28* expression in WT (a) or Δ*rstA* (b) in DMEM and DMEM supplemented with 2 mM EGTA (*n* = 3). (c) Transcriptional activity (*β*-galactosidase units) of the regulatory region and promoter region of *tolC28* in *lacZ* fusion constructs within WT and Δ*rstA* in DMEM and DMEM containing 2 mM EGTA (*n* = 3). (d) Adherence capacity of WT and Δ*rstA* strains to Caco-2 cells in DMEM and DMEM supplemented with 2 mM EGTA (*n* = 3). (f) Comparison of intestinal colonization by WT and Δ*rstA* strains in the distal colon of mice with normal or low-calcium diet for 7days before infection (*n* = 6). Two-tailed unpaired Student's t tests (a‒d) or two-sided Mann‒Whitney U tests (e) were used to calculate *P* values. *P < 0.05, **P < 0.01, ***P < 0.001, n.s., not significant.

To investigate whether RstA directly regulates OI-28, the in-silico promoter analysis of the upstream region of OI-28 was analyzed by using BPROM. The analysis identified a σ70-type promoter containing recognizable −35 (ATGATG) and −10 (GTGATTC T) motifs located upstream of *tolC28* (LDF = 7.02). Then, the RstA box(5-TAATTCAAATTACC-3) were mapped to the putative promoter region and upstream regulatory region. The results showed that the predicted core promoter (−35/−10) does not overlap the putative RstA binding site; the RstA motif resides upstream in the regulatory region (Figure 1b). ChIP-qPCR was further used to confirm the binding of RstA to the regulatory region of *tolC28* in vivo. The results revealed that regulatory region of *tolC28* was enriched approximately 6-fold in RstA-ChIP samples compared with mock-ChIP samples (Figure 4c). Conversely, the fold enrichment of the coding region of *rpoS* (negative control) did not differ between the RstA-ChIP and mock-ChIP samples (Figure 4c). Next, the binding of RstA to regulatory region of *tolC28* in vitro was analyzed via electrophoretic mobility shift assays (EMSAs). The EMSA results revealed considerable shift in the migration of the bands for regulatory region of *tolC28* and P*asr* with increasing concentrations of RstA (Figure 4d, e), whereas no shift in migration bands was observed for *rpoS* (Figure 4f). These results confirmed that RstA directly binds to regulatory region of *tolC28*. To identify the RstA binding site in the regulatory region of *tolC28*, sequence analysis revealed a potential RstA box in *E. coli* (5-TAATTCAAATTACC-3) from −294 to −280 bp. To further determine whether potential RstA boxes are important for binding to RstA, we performed EMSA using a regulatory region of *tolC28*-1 DNA fragment (without the potential RstA box) under the same conditions (Figure 5g). The results revealed that the regulatory region of *tolC28*-1 DNA fragment was unable to bind RstA, confirming that the potential RstA box is crucial for the ability of RstA to bind regulatory region of *tolC28*.

Together, these data indicate that RstA promotes the expression of OI-28 by directly binding to the regulatory region.

### Extracellular Ca^2+^ promotes OI‑28 expression and adherence via RstA.

RTX adhesins depend on extracellular Ca^2+^ for proper folding and activity.^14^ The RstAB TCS functions as a virulence regulator in EHEC O157:H7 and directly binds the OI 28 regulatory region. Therefore, we hypothesized that luminal Ca^2+^ serves as a host-derived signal that is sensed by RstAB to induce OI-28 expression and promote early attachment of EHEC O157:H7.

To test this hypothesis, we first established defined calcium conditions. DMEM, which falls within the physiological range of luminal Ca^2+^ (approximately 2 mM), was used as a baseline. Low-calcium conditions were generated by adding 2 mM EGTA to chelate free Ca^2+^ (EGTA medium). We first analyzed the expression of the T1SS genes (*hlyB28*, *hlyD28*, and *tolC28*) and the RTX genes (*rtxA28* and *rtxB28*) in the WT via qRT-PCR. Compared with that in EGTA medium, the expression of OI-28 in WT media increased approximately 2.27-3.5-fold in DMEM (Figure 5a). Promoter activity assays with the regulatory region of *tolC28-lacZ* reporter confirmed that *β*-galactosidase activity was greater in DMEM and lower in EGTA medium (Figure 5c). However, neither the qRT-PCR nor the regulatory region of *tolC28-lacZ* reporter readout responded to the Ca^2+^ concentration in Δ*rstA* (Figure 5b, c). Next, adherence assays were performed. At 3 h post-infection, compared with DMEM, EGTA significantly reduced WT attachment (Figure 5d). In contrast, Δ*rstA* exhibited reduced adherence, which did not improve upon EGTA supplementation (Figure 5d). These results indicate that Ca^2+^ enhances T1SS-dependent adherence in an RstA-dependent manner. In a murine model, low-calcium diet significantly reduced the early colonization ability of WT, whereas Δ*rstA* showed no corresponding change, which aligns with the in vitro results (Figure 5e). These findings indicate that intestinal Ca^2+^ availability promotes EHEC O157:H7 colonization through RstA.

Together, these results support a model in which extracellular Ca^2+^ is sensed via RstAB to increase OI-28 expression, thereby promoting T1SS assembly and Ca^2+^-dependent folding of RTX adhesins. This coordination strengthens early epithelial adherence and promotes colonization.

### The T1SS is widespread in different pathogenic *E. coli* strains

**Figure 6.**
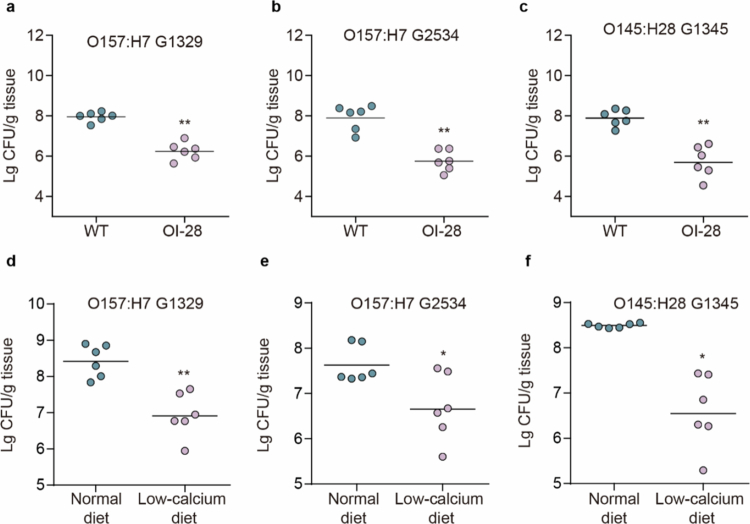
OI‑28 contributes to colonization across pathogenic *E. coli* strains. (a-c) Comparison of intestinal colonization by EHEC O157:H7 G1329 (a), EHEC O157:H7 G2534 (b), EHEC O145:H28 G1345(c) and corresponding mutant strains in the mouse distal colon. Data points represent individual mice; horizontal bars denote medians (*n* = 6). (d-f) Comparison of intestinal colonization by EHEC O157:H7 G1329 (d), EHEC O157:H7 G2534 (e), EHEC O145:H28 G1345(f) in the distal colon of mice with normal or low-calcium diet for 7days before infection(*n* = 6). Two-sided Mann‒Whitney U tests (a-f) were used to calculate *P* values. *P < 0.05, **P < 0.01, ***P < 0.001, n.s., not significant.

On the basis of previous comparative genomics analysis, we found that OI-28 is conserved in several pathogenic strains, including EHEC O157:H7 and EHEC O145:H28 (Figure 1a). To confirm the potential function of OI-28 in these strains, OI-28 deletion strains were constructed in EHEC O157:H7 G1329, EHEC O157:H7 G2534 and O145:H28 G1345. Then, mouse colonization assays were performed. Compared with the WT strains, all these mutants displayed markedly reduced colonization capacities in the colon (Figure 6a-c). These results demonstrated that OI-28-mediated in vivo colonization reflects a general, conserved mechanism in multiple pathogenic *E. coli* lineages.

To further determine whether a low-calcium diet can broadly affect EHEC colonization, we conducted mouse colonization assays under calcium supplementation and a normal diet. The results showed that, in these strains, normal calcium diet significantly enhanced colonic colonization compared with the low-calcium diet (Figure 6d-f). These findings suggest that dietary calcium broadly promotes EHEC colonization in vivo and may enhance the OI‑28-dependent fitness advantages of EHEC during infection.

Collectively, these results demonstrate that the T1SS, a one-step, trans-envelope secretion machine, is conserved and functionally linked to virulence across pathogenic *E. coli*. The concordance between genomic conservation and functional readouts across diverse strains supports the T1SS as a widely utilized, virulence-associated secretion system in pathogenic *E. coli* and highlights limiting calcium intake as a potential anti-virulence strategy.

## Discussion

**Figure 7.**
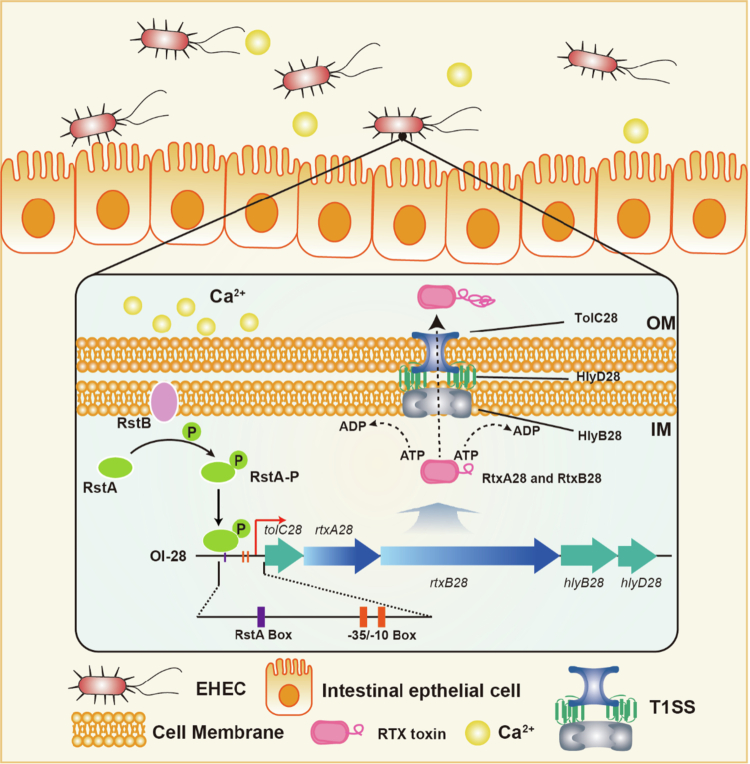
RstA‑mediated regulation of the OI‑28‑encoded T1SS and RTX toxin secretion in EHEC. The OI‑28 encodes a T1SS(*hlyB28*, *hlyD28*, *tolC28*) that secretes RTX toxins (*rtxA28*, *rtxB28*) in a Ca²⁺‑dependent manner. Phosphorylated RstA activates transcription of OI‑28 genes, promoting RTX secretion and EHEC adhesion to intestinal epithelial cells.

EHEC O157:H7 is an important human pathogen that is responsible for diarrheal disease, hemorrhagic colitis, and, in severe cases, hemolytic uremic syndrome.^1^ However, the pathogenic mechanisms of EHEC O157:H7 are not fully understood. In this study, we identified OI-28 as a conserved pathogenic island in EHEC O157:H7, which provides novel insights into the molecular pathogenesis of EHEC O157:H7. The OI-28-encoded T1SS and RTX toxins are essential for the initial adherence and intestinal colonization of EHEC O157:H7. Furthermore, we found that RstA directly binds the OI‑28 regulatory region and activates its transcription by sensing extracellular Ca^2+^. Given the evolutionary conservation of OI‑28 and similar impacts between different EHEC O157:H7 strains, these findings support a general mechanism in which the T1SS exports RTX adhesins to promote EHEC O157:H7 attachment and colonization (Figure 7).

The T1SS is a one-step secret machine that consists of an inner-membrane ABC transporter, a periplasm-spanning membrane fusion protein, and an outer-membrane protein, allowing large adhesins with RTX repeats to be directly transported to the cell surface.^34^ In EHEC O157:H7, OI‑28 encodes a complete T1SS, including the ABC transporter, MFP, and outer-membrane protein.^35^ Together with two RTX proteins whose C‑terminal, calcium-binding repeats and Ig-like domains underpin postsecretion folding and host interaction, these proteins are consistent with ABC recognition of a noncleaved C‑terminal signal and assembly of a continuous trans-envelope conduit for export.^36^ Functionally, the OI‑28 T1SS/RTX system is indispensable for early adherence to the epithelium. ΔOI‑28 exhibited markedly reduced adherence at 3 h post infection, a decrease in A/E‑like pedestals, and attenuated intestinal colonization without growth defects. Furthermore, each gene in OI‑28 is required for activity, as single-gene mutants diminish adhesion, pedestal formation, and colonization. In addition, complementation of the ABC transporter, MFP, or OMP restores these phenotypes to WT levels. Domain-focused analyzes further revealed that the RTX Ig-like repeats are required and sufficient for virulence-associated attachment, indicating that the OI‑28 T1SS presents an adhesin during the early stages of infection. These adherences occur independently of the T3SS, indicating the complexity of EHEC O157:H7 colonization and helping explain efficient host attachment and potential therapeutic targets of the T1SS.

Pathogens have developed diverse mechanisms to sense and respond to environmental signals.^37^ TCSs are important for bacteria to regulate gene expression in response to various environmental changes to adapt to a specific niche.^38^ Upon entering the human colon, TCS-mediated signaling pathways are integral to the regulation of virulence factors such as toxins and adhesins, which are crucial for establishing infection in EHEC O157:H7.^22^^,^^39^ In this study, we found that RstA directly regulates the expression of OI‑28 T1SS‑RTX during colonization. Deletion of *rstA* reduces the expression levels of the OI‑28‑encoded T1SS components(*hlyB28*, *hlyD28*, and *tolC28*) and RTX genes (*rtxA28* and *rtxB28*) relative to those of the WT. Furthermore, ChIP‑qPCR and EMSA confirmed the direct binding of the RstA box in the regulatory region required for the interaction. Importantly, Ca^2+^ emerges as a host-derived environmental cue that influences secretion with adhesin functionality in this pathway.^40^ RTX proteins require extracellular Ca^2+^ for rapid post-secretion folding into their functional conformations, enabling host-cell adhesion.^41^ Consistent with this, we showed that physiological levels of extracellular Ca^2+^ enhance OI-28 transcription and T1SS-dependent adherence in an RstA-dependent manner, whereas Ca^2+^ chelation diminishes both activity and attachment. In vivo, dietary Ca^2+^ supplementation increases early colonization by WT but not Δ*rstA*, indicating that RstA is necessary to transduce Ca^2+^ availability to OI-28 activation. Together, these results support a model in which RstA senses or integrates Ca^2+^-linked signals to upregulate OI-28, thereby coordinating T1SS assembly with Ca^2+^-dependent folding of RTX adhesins to promote initial adhesion during distinct stages of intestinal colonization.

The evolution of different EHEC O157:H7 lineages involves repeatedly obtaining a core set of virulence factors via horizontal gene transfer to generate the same pathogenic features, the chief of which is the independent acquisition of the LEE pathogenicity island encoding the T3SS.^23^ Comparative genomics revealed that OI‑28 is highly conserved across EHEC O157:H7, EHEC O145:H28 and other pathogenic *E. coli* lineages, suggesting strong environmental selection for its maintenance and functional importance in disease. In this study, we confirmed the indispensability of OI‑28 for full virulence in a murine model of different pathogenic *E. coli* strains, including EHEC O157:H7 and EHEC O145:H28. Comparative genomics further revealed that OI‑28 is conserved across pathogenic *E. coli* lineages and confers a broad colonization advantage, indicating that the T1SS/RTX system is a general, T3SS‑independent virulence pathway and a promising antivirulence target. The combination of convergent genomic phenotypes also implies sustained selection pressure for OI‑28 and is consistent with potential dissemination via horizontal gene transfer, a scenario that would help explain its prevalence among virulent lineages and its contribution to outbreak‑associated strains.

In summary, we identified OI‑28 as a conserved pathogenicity island that encodes a complete T1SS, collectively driving a T3SS‑independent adhesion pathway in EHEC O157:H7. Future work will clarify the interaction between the T3SS and T1SS across infection stages. These findings will enable strategies that block the function of the T1SS and contribute to broader efforts to develop precision therapeutics against microbial pathogens.

## Materials and methods

### Strains and plasmids

The bacterial strains and plasmids used in this study are listed in Supplementary Tables 1 and 2.

### Variant calling strategies and phylogenetic analysis

Snippy (v4.6.0) (Seemann; GitHub: https://github.com/tseemann/snippy) was used to identify single-nucleotide polymorphisms (SNPs) by mapping sequencing reads from 2,122 samples (listed in Supplementary Table 3) to the reference genome, followed by variant calling with FreeBayes using default parameters. Core SNPs with high quality were extracted for downstream analyzes. Parsnp1 (v2.1.4) was used to construct a genome-wide alignment of the samples against the reference genome, focusing on conserved core regions. The resulting core-genome SNP alignment was then used to infer a maximum-likelihood phylogenetic tree.^42^

### Detailed domain structure analysis and subcellular localization and potential promoter prediction

The conserved domain architecture of the proteins was analyzed using the NCBI Conserved Domain Database (CDD; https://www.ncbi.nlm.nih.gov/Structure/cdd/). Each predicted amino acid sequence was submitted to these databases to identify conserved motifs and functional domains. Protein schematics were generated based on the consensus domain architecture obtained from these analyzes.

The subcellular localization of the proteins was predicted using PSORTb v3.0 (https://www.psort.org/psortb/). The complete amino acid sequences were analyzed under the default parameters for Gram‑negative bacteria. Localization scores were obtained for the cytoplasmic, inner‑membrane, periplasmic, outer‑membrane, and extracellular compartments. The final localization was determined based on the highest prediction score.

The upstream region (320 bp) of *tolC28* was analyzed in silico to predict potential promoter elements using BPROM (SoftBerry; http://www.softberry.com/berry.phtml?topic=index&group=programs&subgroup=gfindb). This program predicts bacterial σ70‑dependent promoters based on the presence of −35 and −10 consensus motifs and calculates a linear discriminant function (LDF) score to estimate promoter strength. Sequences with an LDF > 0 are considered likely promoters, and higher scores indicate stronger promoter probability. To further characterize transcriptional control, the predicted RstA binding site identified in previous assays was mapped onto the same upstream region and compared with the position of the predicted −35/−10 elements.

### Cell lines and animals

Caco-2 cells were obtained from the Shanghai Institute of Biochemistry and Cell Biology of the Chinese Academy of Sciences (Shanghai, China). The cells were cultured in DMEM supplemented with 10% fetal bovine serum (FBS) at 37 °C under 5% CO_2_. Six-week-old female BALB/c mice were purchased from Beijing Vital River Laboratory Animal Technology (Beijing, China) and housed at 37 °C under a 12 h light/dark cycle.

### Mutants construction and complementation

The ΔO1-28, Δ*tolC28*, Δ*rtxA28*, Δ*rtxB28*, Δ*hlyB28*, Δ*hlyD28*, and Δ*rstA* mutants were constructed via the *λ*-Red recombination method. The complementary strains (Δ*tolC28*+, Δ*rtxA28*+, Δ*rtxB28*+, Δ*hlyB28*+, Δ*hlyD28*+, and Δ*rstA*+) were constructed by inserting the corresponding gene into the plasmid pTrc99a. For RstA purification, *rstA* was cloned and inserted into the plasmid pET28a. The LacZ reporter strain was generated by cloning the regulatory region and promoter region of *tolC28* into the pHRP309 plasmid. All the plasmids were introduced into the strains via electroporation. When needed, antibiotics were added at the following final concentrations: 100 μg/ml ampicillin, 25 μg/ml chloramphenicol, 50 μg/ml kanamycin, and 25 μg/ml gentamicin.

### Bacterial growth curves

Overnight bacterial cultures were washed with sterile phosphate-buffered saline (PBS) and diluted 1:1000 in DMEM or LB broth in 96-well plates. Growth was monitored via a multifunctional microplate reader (TECAN Spark, Shanghai, China) at 37 °C with shaking and measured at OD_600_ for 24 h. Three independent experiments were performed.^43^

### Cell adhesion assays

Prior to infection, Caco-2 cells were seeded in 6-well plates and incubated for 24 h. The cells were then washed, and the culture medium was replaced with serum-free DMEM. When necessary, 2 mM EGTA was added to DMEM to chelate free Ca^2+^ before infection. Mid-log phase bacteria were added to the monolayers (MOI = 10) and incubated for 3, 6 and 9 h at 37 °C with 5% CO_2_. To ensure comparability among samples, the initial bacterial inoculum used for each well was quantified to confirm equal input across all groups, thereby excluding variation in starting bacterial numbers as a confounding factor. Following infection, the cells were washed with PBS and lysed in 0.1% sodium dodecyl sulfate. Serial dilutions of the lysates were plated on LB agar containing the appropriate antibiotics. Colony-forming units (CFU)/ml were enumerated to determine attachment efficiency. The assay included at least three independent biological replicates.

### Fluorescent actin staining (FAS)

FAS assays were performed according to previously described methods.^44^ Sterile coverslips were placed in 6-well plates prior to seeding Caco-2 cells. The cell culture conditions and infection procedures were identical to those used in the adhesion assays. After 3 h of coincubation, the coverslips were washed with PBS, fixed with 4% paraformaldehyde, and permeabilized with 0.1% Triton X-100. Actin filaments were visualized by staining with fluorescein isothiocyanate-labeled phalloidin (Sigma-Aldrich, Shanghai, China), whereas host nuclei and bacteria were stained with propidium iodide (Beyotime, Shanghai, China). For each bacterial strain, at least 50 Caco-2 cells were examined to quantify the of A/E lesions.

### Mouse infection assays

Mouse infection experiments were carried out as previously reported with minor adjustments.^45^ When required, mice received either a low-calcium diet (0.1% Ca²⁺) or a normal-calcium diet (1.0-1.5% Ca²⁺) for 7 days before infection. The six-week-old female BALB/c mice were orally administered a cocktail of four antibiotics, ampicillin, neomycin, metronidazole, and vancomycin (Sigma-Aldrich), for 3 days to deplete gut microbiota (5 mg each antibiotic per mouse per day) and fasted for 22 h before infection. The bacterial cultures were pelleted via centrifugation, washed, and resuspended in PBS. Each animal received an oral injection of 1 × 10^9^ CFU in 100 μL of PBS. At 8 h postinfection, the mice were anesthetized and euthanized via cervical dislocation. The distal colon tissue was excised, and the luminal contents were removed and weighed. The distal colon tissue and luminal contents samples were homogenized in PBS, serially diluted, and plated on LB agar containing the corresponding antibiotics for CFU enumeration.

To evaluate the severity of infection, mouse body weight was monitored daily from the day of infection (day 0) to the end of the experiment (day 7). Weight change was expressed as a percentage relative to the initial body weight at day 0. Survival of each group was also recorded for 7 days post‑infection. Survival was monitored daily at consistent time points throughout the study. The statistical significance between groups was evaluated using the log‑rank (Mantel–Cox) test.

### RNA extraction and quantitative RT-PCR

Overnight LB cultures were diluted 1:100 in DMEM and incubated to the exponential phase. The cells were collected, and total RNA was extracted with TRIzol reagent (Invitrogen; 5596026). The RNA quality was verified via gel electrophoresis, and the purity was assessed via a Nanodrop 2000 spectrophotometer. cDNA synthesis was performed via the use of StarScript III RT MasterMix (Genestar, A233) with SYBR Green (Genestar, A304) for quantitative PCR. The reactions were run on an ABI 7500 thermocycler, and gene expression was normalized to that of *rpoA.*^46^ Relative expression levels were calculated via the 2^-ΔΔCt^ method. The data were collected from three biological replicates.

### LacZ fusion assays

Overnight cultures were inoculated into 20 mL of fresh DMEM and grown to the exponential phase. The cells were harvested for *β*-galactosidase activity measurement following Miller’s method.^47^ The results are expressed in Miller units, and each assay included at least three independent biological replicates.

### Chromatin immunoprecipitation-qPCR (ChIP-qPCR)

The ChIP assay was conducted following previously described.^48^ Overnight cultures of bacterial strains were grown in LB broth at 37 °C with shaking at 180 rpm. These cultures were then inoculated into 200 mL fresh LB medium at a ratio of 1:100 and incubated until the optical density at 600 nm reached 0.6-0.8. Crosslinking was performed by adding 1% formaldehyde at the final concentration (Macklin, F809702) and incubating at room temperature for 25 min without shaking. The reaction was quenched by adding glycine (Macklin, G6197) to a final concentration of 0.5 M, followed by by a 5-min incubation. Cells were harvested by centrifugation (5,000 × g, 5 min, 4 °C) and resuspended in lysis buffer (50 mM Tris-HCl pH 7.5, 100 mM NaCl, 1 mM EDTA, 1 mM PMSF, 20mg/ml lysozyme, 0.5mg/ml RNase), then incubated at 37 °C for 30 min. Chromatin was fragmented by sonication (30% amplitude, 30 s on/30 s off, 9 cycles) to generate 200-500 bp DNA fragments, and supernatants were collected by centrifugation (12,000 × g, 10 min, 4 °C). Lysates were incubated overnight at 4 °C with an anti-FLAG antibody (Sigma-Aldrich, B3111) to capture the target protein-DNA complexes, followed by incubation with protein A magnetic beads (Invitrogen, 10002D) for 5 h at 4 °C. Non-specific interactions were removed by washing three times with cleaning buffer (100 mM Tris-HCl pH 7.5, 200 mM NaCl, 1 mM EDTA, 2% Triton X-100). DNA-protein complexes were eluted with elution buffer (50 mM Tris-HCl pH 8.0, 10 mM EDTA, 1% SDS) at 65 °C for 30 min. DNA was purified using a DNA Extraction Kit (Sparkjade; AE0101) and subjected to qPCR analysis. Experiment was independently repeated at least three times for statistical analysis.

### Electrophoretic mobility shift assay (EMSA)

EMSA was carried out using purified 6 × His-tagged RstA protein expressed in *E. coli* strain BL21 (DE3). The regulatory regions of *tolC28*, *tolC28-1*, *asr*, and *rpoS* were amplified via PCR and purified with the DNA Extraction Kit (Sparkjade; AE0101). 40 ng of purified DNA fragments and 0-2 μM of purified RstA protein were incubated at 25 °C for 30 min in EMSA binding buffer (20 mM Tris-HCl pH 7.5, 50 mM KCl, 1 mM DTT, and 10% glycerol [v/v]) to a final volume of 20 μl. The resulting protein-DNA complexes were resolved on native polyacrylamide gels at 4 °C under 80 V/cm. Gels were stained with 0.1% GelRed for 10 min, and shifted bands were visualized under UV transillumination.

## Supplementary Material

Revised_Supplementary_InformationCleanVersion.docx Revised_Supplementary_InformationCleanVersion.docx

Supplementary_Table_1_and_2CleanVersion.docx Supplementary_Table_1_and_2CleanVersion.docx

Supplementary Table 3.xlsx Supplementary Table 3.xlsx

## Data Availability

All genome sequence sources are listed in the Materials and Methods and are publicly available via the NCBI Datasets portal (https://www.ncbi.nlm.nih.gov/datasets/genome/?taxon=562). The GenBank accessions and assembly identifiers used in this study are provided in Supplementary Table 3. All data supporting the findings are presented in the figures and described in the text.

## Abbreviations

A/E lesion: Attaching and effacing lesion
ChIP-Qpcr: Chromatin immunoprecipitation‒quantitative polymerase chain reaction
TCS: two-component system
DMEM: Dulbecco’s modified Eagle medium
EHEC: Enterohemorrhagic *Escherichia coli*
EMSA: Electrophoretic mobility shift assay
FAS: Fluorescent actin staining
HC: Hemorrhagic colitis
HUS: Hemolytic uremic syndrome
LB: Luria-Bertani (broth)
LEE: Locus of enterocyte effacement
MFP: Membrane fusion protein
OMP: Outer membrane protein
RTX: Repeats‑in‑toxin
T1SS: Type I secretion system
T3SS: Type III secretion system
WT: Wild type

## References

[1] Park H, Lee K, Shin H, Holzapfel WH. Autoinducer-2 Quorum sensing influences viability of *Escherichia coli* O157:H7 under osmotic and in vitro gastrointestinal stress conditions. Front Microbiol. 2017;8:1077. doi: 10.3389/fmicb.2017.01077. 28659895 PMC5468425

[2] Brandelli JR, Griener TP, Laing A, Mulvey G, Armstrong GD. The effects of Shiga toxin 1, 2 and their subunits on Cytokine and Chemokine expression by human macrophage-like THP-1 Cells. Toxins (Basel). 2015;7(10):4054–4066. doi: 10.3390/toxins7104054. 26473922 PMC4626720

[3] SY Yang, Yoon KS. Quantitative microbial risk assessment of listeria monocytogenes and Enterohemorrhagic *Escherichia coli* in yogurt. Foods. 2022;11(7). 10.3390/foods11070971 PMC8997960 35407058

[4] Bai Z, Zhang S, Wang X, Aslam MZ, Li H, Dong Q. Genotyping based on CRISPR loci diversity and pathogenic potential of diarrheagenic escherichia coli. Front Microbiol. 2022;13:852662. doi: 10.3389/fmicb.2022.852662. 35308371 PMC8924505

[5] Li L, Liu Y, Wang J, Xiang B, Qin J, Yao T, Wu P, Zhang J, Xu Y, Ma G, et al. Microbiota-derived succinate promotes enterohaemorrhagic escherichia coli virulence via lysine succinylation. Nat Microbiol. 2025;10(3):749–764. doi: 10.1038/s41564-025-01931-x. 39891012

[6] Gelalcha BD, Brown SM, Crocker HE, Agga GE, Kerro Dego O. Regulation mechanisms of virulence genes in Enterohemorrhagic *escherichia coli*. Foodborne Pathog Dis. 2022;19(9):598–612. doi: 10.1089/fpd.2021.0103. 35921067

[7] Kumar A, Sperandio V. Indole signaling at the host-microbiota-pathogen interface. mBio. 2019;10(3), 10.1128/mBio.01031-19. PMC6550529 31164470

[8] Vinh PT, Shinohara Y, Yamada A, Duc HM, Nakayama M, Ozawa T, Sato J, Masuda Y, Honjoh K, Miyamoto T. Baicalein inhibits Stx1 and 2 of EHE: effects of baicalein on the cytotoxicity, production, and secretion of Shiga toxins of Enterohaemorrhagic escherichia coli. Toxins (Basel). 2019;11(9):505. doi: 10.3390/toxins11090505. 31470657 PMC6784239

[9] McWilliams BD, Torres AG. Enterohemorrhagic *escherichia coli* adhesins. Microbiol Spectr. 2014;2(3), 10.1128/microbiolspec.EHEC-0003-2013. 26103974

[10] Yu M, Chen Y, Cao X, Su S, Wu J, Sun L, Lu Q, Zhang C, Deng Z, Li J, et al. A systematic survey of type I secretion systems and their substrate proteins in Salmonella. Virulence. 2025;16(1):2533414. doi: 10.1080/21505594.2025.2533414. 40662566 PMC12269683

[11] Pourhassan ZN, Cui H, Muckhoff N, Davari MD, Smits SHJ, Schwaneberg U, Schmitt L. A step forward to the optimized HlyA type 1 secretion system through directed evolution. Appl Microbiol Biotechnol. 2023;107(16):5131–5143. doi: 10.1007/s00253-023-12653-7. 37405436 PMC10386944

[12] Masi M, Wandersman C. Multiple signals direct the assembly and function of a type 1 secretion system. J Bacteriol. 2010;192(15):3861–3869. doi: 10.1128/JB.00178-10. 20418390 PMC2916380

[13] Smith TJ, Font ME, Kelly CM, Sondermann H, O'Toole GA. An N-terminal retention module anchors the giant adhesin LapA of pseudomonas fluorescens at the cell surface: a novel subfamily of type I secretion systems. J Bacteriol. 2018;200(8), 10.1128/JB.00734-17. PMC5869472 29437852

[14] Linhartova I, Linhartová I, Bumba L, Mašín J, Basler M, Osička R, Kamanová J, Procházková K, Adkins I, Hejnová-Holubová J, et al. RTX proteins: a highly diverse family secreted by a common mechanism. FEMS Microbiol Rev. 2010;34(6):1076–1112. doi: 10.1111/j.1574-6976.2010.00231.x. 20528947 PMC3034196

[15] Chenal A, Guijarro JI, Raynal B, Delepierre M, Ladant D. RTX calcium binding motifs are intrinsically disordered in the absence of calcium: implication for protein secretion. J Biol Chem. 2008;284(3):1781–1789. doi: 10.1074/jbc.M807312200. 19015266

[16] Welch RA. RTX toxin structure and function: a story of numerous anomalies and few analogies in toxin biology. Curr Top Microbiol Immunol. 2001;257:85–111. doi: 10.1007/978-3-642-56508-3_5. 11417123

[17] González-Bullón D, Uribe K, Largo E, Guembelzu G, García-Arribas AB, Martín C, Ostolaza H. Membrane permeabilization by Bordetella adenylate cyclase toxin involves pores of tunable size. Biomolecules. 2019;9(5):183. doi: 10.3390/biom9050183. 31083482 PMC6572617

[18] Griessl MH, Schmid B, Kassler K, Braunsmann C, Ritter R, Barlag B, Stierhof Y, Sturm KU, Danzer C, Wagner C, et al. Structural insight into the giant Ca²⁺-binding adhesin SiiE: implications for the adhesion of Salmonella enterica to polarized epithelial cells. Structure. 2013;21(5):741–752. doi: 10.1016/j.str.2013.02.020. 23562396

[19] Justice SS, Hunstad DA. UPEC hemolysin: more than just for making holes. Cell Host Microbe. 2012;11(1):4–5. doi: 10.1016/j.chom.2012.01.001. 22264508 PMC3267088

[20] Wick LM, Qi W, Lacher DW, Whittam TS. Evolution of genomic content in the stepwise emergence of *Escherichia coli* O157:H7. J Bacteriol. 2005;187(5):1783–1791. doi: 10.1128/JB.187.5.1783-1791.2005. 15716450 PMC1064018

[21] Han J, Gao X, Luo X, Zhu L, Zhang Y, Dong P. The role of PhoP/PhoQ system in regulating stress adaptation response in escherichia coli O157:H7. Food Microbiol. 2023;112:104244. doi: 10.1016/j.fm.2023.104244. 36906298

[22] Sun H, Huang D, Pang Y, Chen J, Kang C, Zhao M, Yang B. Key roles of two-component systems in intestinal signal sensing and virulence regulation in enterohemorrhagic Escherichia coli. FEMS Microbiol Rev. 2024;48(6), 10.1093/femsre/fuae028. PMC11644481 39537200

[23] Liu B, Yang B, Wang Q, Qin J, Zhao K, Li F, Feng X, Wu P, Zhu S. Escherichia coli O157:H7 senses microbiota-produced riboflavin to increase its virulence in the gut. Proc Natl Acad Sci U S A. 2022;119(48):e2212436119. doi: 10.1073/pnas.2212436119. 36409903 PMC9860305

[24] Liu Y, Han R, Wang J, Yang P, Boyle JP. Magnesium sensing regulates intestinal colonization of Enterohemorrhagic escherichia coli O157:H7. mBio. 2020;11(6), 10.1128/mBio.02470-20. PMC7667037 33173003

[25] Hughes DT, Clarke MB, Yamamoto K, Rasko DA, Sperandio V. The QseC adrenergic signaling cascade in Enterohemorrhagic *E. coli* (EHEC). PLoS Pathog. 2009;5(8):e1000553. doi: 10.1371/journal.ppat.1000553. 19696934 PMC2726761

[26] Thomassin J-L, Giannakopoulou N, Zhu L, Gross J, Salmon K, Leclerc J, Daigle F, Le Moual H, Gruenheid S, Bäumler AJ. The CpxRA two-component system is essential for citrobacter rodentium virulence. Infect Immun. 2015;83(5):1919–1928. doi: 10.1128/IAI.00194-15. 25712925 PMC4399039

[27] Liu Y, Li S, Wang P, Ding P, Yang P, Xu T, Xiong Y. RstA, a two-component response regulator, plays important roles in multiple virulence-associated processes in enterohemorrhagic Escherichia coli O157:H7. Gut Pathog. 2019;11:53. doi: 10.1186/s13099-019-0335-4. 31695752 PMC6824119

[28] Guragain M, King MM, Williamson KS, Pérez-Osorio AC, Akiyama T, Khanam S, Patrauchan MA, Franklin MJ, O'Toole GA. The Pseudomonas aeruginosa PAO1 two-component regulator CarSR regulates calcium homeostasis and calcium-induced virulence factor production through its regulatory targets CarO and CarP. J Bacteriol. 2016;198(6):951–963. doi: 10.1128/JB.00963-15. 26755627 PMC4772601

[29] Jimenez AG, Ellermann M, Abbott W, Sperandio V. Diet-derived galacturonic acid regulates virulence and intestinal colonization in enterohaemorrhagic *escherichia coli* and *citrobacter rodentium*. Nat Microbiol. 2019;5(2):368–378. doi: 10.1038/s41564-019-0641-0. 31873206 PMC6992478

[30] Alav I, Kobylka J, Kuth MS, Pos KM, Picard M, Blair JMA, Bavro VN. Structure, Assembly, and function of tripartite efflux and type 1 secretion systems in gram-negative bacteria. Chem Rev. 2021;121(9):5479–5596. doi: 10.1021/acs.chemrev.1c00055. 33909410 PMC8277102

[31] Pourhassan NZ, Smits SHJ, Ahn JH, Schmitt L. Biotechnological applications of type 1 secretion systems. Biotechnol Adv. 2021;53:107864. doi: 10.1016/j.biotechadv.2021.107864. 34767962

[32] Motlova L, Klimova N, Fiser R, Sebo P, Bumba L. Continuous assembly of β-roll structures is implicated in the type I-dependent secretion of large repeat-in-toxins (RTX) proteins. J Mol Biol. 2020;432(20):5696–5710. doi: 10.1016/j.jmb.2020.08.020. 32860773

[33] Guo S, Vance TDR, Stevens CA, Voets I, Davies PL. RTX Adhesins are key bacterial surface megaproteins in the formation of Biofilms. Trends Microbiol. 2019;27(5):453–467. doi: 10.1016/j.tim.2018.12.003. 30658900

[34] Holland IB, Schmitt L, Young J. Type 1 protein secretion in bacteria, the ABC-transporter dependent pathway (review). Mol Membr Biol. 2005;22(1-2):29–39. doi: 10.1080/09687860500042013. 16092522

[35] Reid SD, Herbelin CJ, Bumbaugh AC, Selander RK, Whittam TS. Parallel evolution of virulence in pathogenic *escherichia coli*. Nature. 2000;406(6791):64–67. doi: 10.1038/35017546. 10894541

[36] Li S, Liu H, Qiu J. Emerging insights into the type I secretion system: a key player in *Salmonella* virulence and host-pathogen interactions. mBio. 2025. e0165525. 40981436 10.1128/mbio.01655-25 PMC12607715

[37] Balasubramanian D, López-Pérez M, Grant T-A, Ogbunugafor CB, Almagro-Moreno S. Molecular mechanisms and drivers of pathogen emergence. Trends Microbiol. 2022;30(9):898–911. 35248462 10.1016/j.tim.2022.02.003

[38] Alvarez AF, Georgellis D. Environmental adaptation and diversification of bacterial two-component systems. Curr Opin Microbiol. 2023;76:102399. doi: 10.1016/j.mib.2023.102399. 39399893

[39] Tiwari S, Jamal SB, Hassan SS, Carvalho PVSD, Almeida S, Barh D, Ghosh P, Silva A, Castro TLP, Azevedo V. Two-component signal transduction systems of pathogenic bacteria as targets for antimicrobial therapy: an overview. Front Microbiol. 2017;8:1878. doi: 10.3389/fmicb.2017.01878. 29067003 PMC5641358

[40] Wanford JJ, Odendall C. Ca^2+^-calmodulin signalling at the host-pathogen interface. Curr Opin Microbiol. 2023;72:102267. doi: 10.1016/j.mib.2023.102267. 36716574

[41] Baumann U. Structure-function relationships of the repeat domains of RTX toxins. Toxins (Basel). 2019;11(11):657. doi: 10.3390/toxins11110657. 31718085 PMC6891781

[42] Treangen TJ, Ondov BD, Koren S, Phillippy AM. The harvest suite for rapid core-genome alignment and visualization of thousands of intraspecific microbial genomes. Genome Biol. 2014;15(11):524. doi: 10.1186/s13059-014-0524-x. 25410596 PMC4262987

[43] Nguyen YN, Sheng H, Dakarapu R, Falck JR, Hovde CJ, Sperandio V, Bäumler AJ. The acyl-homoserine lactone synthase YenI from Yersinia enterocolitica modulates virulence gene expression in enterohemorrhagic escherichia coli O157:H7. Infect Immun. 2013;81(11):4192–4199. doi: 10.1128/IAI.00889-13. 23980115 PMC3811827

[44] Liu Y, Yang P, Wang T, Chang Z, Li W, Wu J, Huang D, Jiang L. LysR-type transcriptional regulator OvrB encoded in O island 9 drives enterohemorrhagic escherichia coli O157:H7 virulence. Virulence. 2019;10(1):783–792. doi: 10.1080/21505594.2019.1661721. 31502495 PMC6768210

[45] Wu P, Wang Q, Yang Q, Feng X, Liu X, Sun H, Yan J, Kang C. A novel role of the two-component system response regulator UvrY in enterohemorrhagic escherichia coli O157:H7 pathogenicity regulation. Int J Mol Sci. 2023;24(3):2297. doi: 10.3390/ijms24032297. 36768620 PMC9916836

[46] Hernandez-Doria JD, Sperandio V. Bacteriophage transcription factor cro regulates virulence gene expression in Enterohemorrhagic esche*richia coli*. Cell Host Microbe. 2018;23(5):607–617.e6. doi: 10.1016/j.chom.2018.04.007. 29746832 PMC5982111

[47] Galvis F, Ageitos L, Rodríguez J, Jiménez C, Barja JL, Lemos ML, Balado M. Vibrio neptunius produces piscibactin and amphibactin and both siderophores contribute significantly to virulence for clams. Front Cell Infect Microbiol. 2021;11:750567. doi: 10.3389/fcimb.2021.750567. 34760718 PMC8573110

[48] Liu Y, Wu J, Li F, Xuan L, Wang Q, Chen X, Sun H, Jin C, Huang D, Tang G. Vibrio cholerae virulence is blocked by chitosan oligosaccharide-mediated inhibition of ChsR activity. Nat Microbiol. 2024;9(11):2909–2922. doi: 10.1038/s41564-024-01823-6. 39414933

