# Supplementary material for: O‑Island 28 encodes a type I secretion and RTX adhesion system regulated by RstA and required for early EHEC O157:H7 adherence
Source: Gut Microbes. 2025 Dec 27;18(1):2609461. doi: 10.1080/19490976.2025.2609461 (PMC12758282; doi:10.1080/19490976.2025.2609461)
Supplement: Revised_Supplementary_InformationCleanVersion.docx [file KGMI_A_2609461_SM9071.docx]

**Supplementary Information for**

**O‑Island 28 Encodes a Type I Secretion and RTX Adhesion System Regulated by RstA and Required for Early EHEC O157:H7 Adherence**


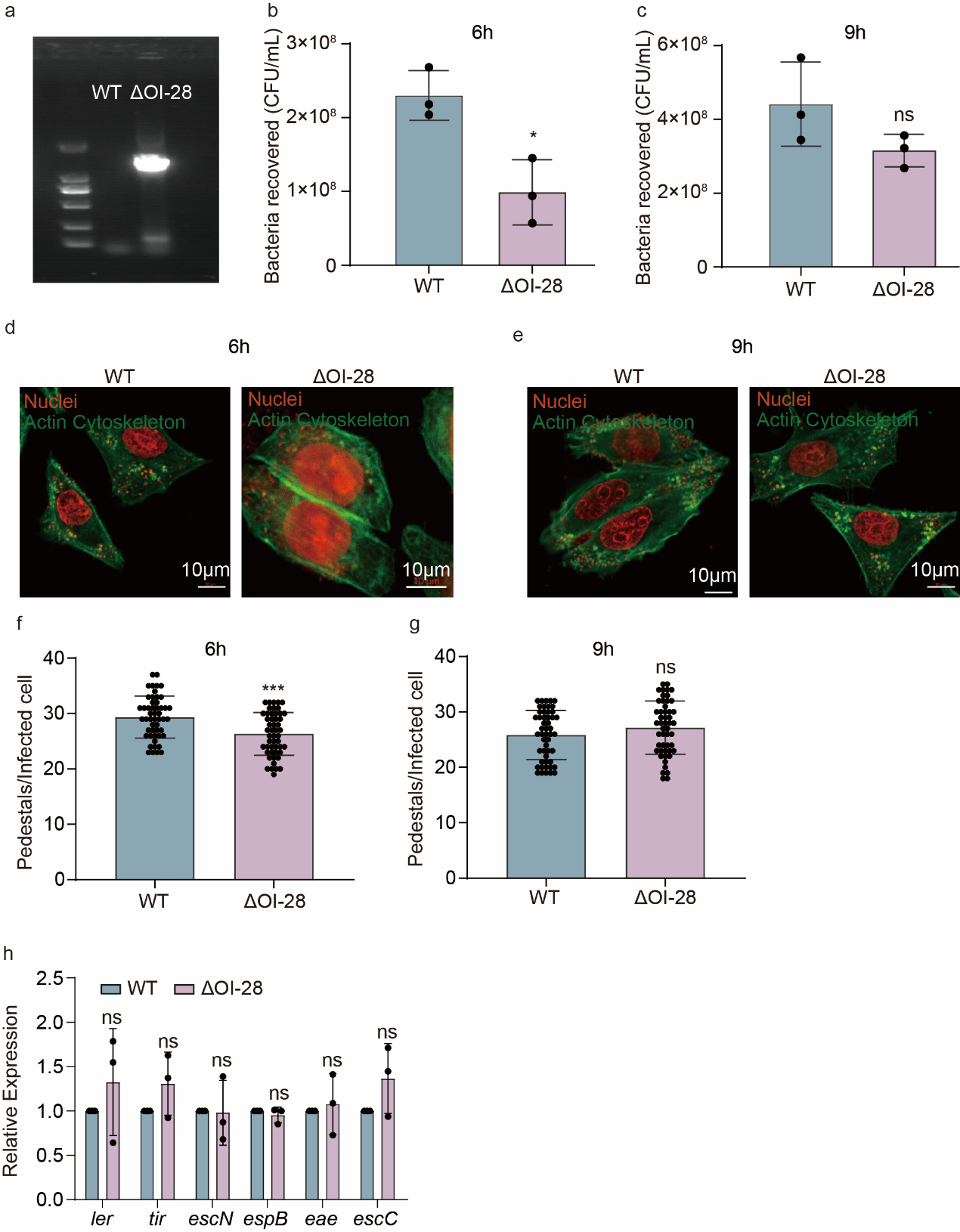


**Fig. S1** (a) PCR analysis confirming successful deletion of the OI‑28 in the mutant strain. (b, c) Adherence of WT and ΔOI‑28 to Caco-2 cells at 6 h(b) and 9 h(c) p.i. (n=3). (d, e) Representative FAS images of WT and ΔOI‑28 strains to Caco-2 cells at 6 h(d) and 9 h(e) p.i. (n=3); nuclei (propidium iodide, red), actin (FITC‑phalloidin, green). Arrowheads indicate pedestals. Scale bar, 10 μm. (f, g) Quantification of pedestals per infected cell of WT and ΔOI‑28 strains to Caco-2 cells at 6 h(f) and 9 h(g) p.i. (n=3), n=50 cells per strain. (h) qRT‒PCR analysis of changes in *ler, tir, escN, espB, eae and escC* expression in the WT and ΔOI‑28 mutant(n=3). Two-tailed unpaired Student's t test (b, c, f, g, h) was used to calculate *P* values. **P*<0.05, ***P*<0.01, ****P*<0.001, n.s., not significant.
