# Supplementary material for: O‑Island 28 encodes a type I secretion and RTX adhesion system regulated by RstA and required for early EHEC O157:H7 adherence
Source: Gut Microbes. 2025 Dec 27;18(1):2609461. doi: 10.1080/19490976.2025.2609461 (PMC12758282; doi:10.1080/19490976.2025.2609461)
Supplement: Supplementary_Table_1_and_2CleanVersion.docx [file KGMI_A_2609461_SM9069.docx]

**Supplementary Table 1**

**Strains and plasmids used in this study.**

| **Strains** | **Genotype or description** | **Source** |
| --- | --- | --- |
| EDL933 | Wild-type EHEC O157:H7 EDL933 | ATCC* |
| G1329 | Wild-type O157:H7 G1329 | Lab collection |
| G2534 | Wild-type O157:H7 G2534 | Lab collection |
| G1345 | Wild-type O145:H28 G1345 | Lab collection |
| BL21(DE3) | *E. coli* BL21(DE3) strain | Lab collection |
| DH5α | *E. coli* DH5α strain | Lab collection |
| Δ*OI-28* | *OI-28* deletion mutant in EDL933 | This work |
| Δ*tolC28* | *tolC28* deletion mutant in EDL933 | This work |
| Δ*rtxA28* | *rtxA28* deletion mutant in EDL933 | This work |
| Δ*rtxB28* | *rtxB28* deletion mutant in EDL933 | This work |
| Δ*hlyB28* | *hlyB28* deletion mutant in EDL933 | This work |
| Δ*hlyD28* | *hlyD28* deletion mutant in EDL933 | This work |
| Δ*tolC28+* | Δ*tolC28* containing pTrc99a-*tolC28* | This work |
| Δ*rtxA28+* | Δ*rtxA28* containing pTrc99a-*rtxA28* | This work |
| Δ*rtxB28+* | Δ*rtxB28* containing pTrc99a-*rtxB28* | This work |
| Δ*hlyB28+* | Δ*hlyB28* containing pTrc99a-*hlyB28* | This work |
| Δ*hlyD28+* | Δ*hlyD28* containing pTrc99a-*hlyD28* | This work |
| H2667 | DH5α containing pTRC99a-*rstA* | This work |
| WT-*lacZ* | EDL933 containing pHRP309-P*z0608*‑*lacZ* | This work |
| Δ*rstA*-*lacZ* | Δ*rstA* containing pHRP309-P*z0608*‑lacZ | This work |
| Δ*rstA+*-*lacZ* | Δ*rstA+* containing pHRP309-P*z0608*‑*lacZ* | This work |
| **Plasmids** | | |
| pKD3 | Containing a chloramphenicol resistance cassette and the flipase recognition sites, CmR | Lab collection |
| pKD46 | Red recombination plasmid, ApR | Lab collection |
| pET28a | T7 expression vector, KmR | Lab collection |
| pTRC99a | Expression vector, ApR | Lab collection |
| pHRP309 | Promoter-reporter vector, CmR | Lab collection |

^*^, ATCC, American Type Culture Collection, Manassas, Virginia, USA.

**Supplementary Table 2**

**Primers used in this study (5'-3').**

| Primers for gene mutation | | | |
| --- | --- | --- | --- |
| *tolC28* | F | CCAGTTAAAATAATGACCTGGAAGGAAGTGACTTATCT GTGTAGGCTGGAGCTGCTTCG | |
| *tolC28* | R | AATCACTTAACAACGGCCCACTATTAATAATGGCAGGA CATATGAATATCCTCCTTAG | |
| *rtxA28* | F | GAGG TTAACGGTGACCGTGTGTG GGATCC GCG GCG GGTACC TAATGGCATTCCTGATAAATGGCGC | |
| *rtxA28* | R | GCGCCATTTATCAGGAATGCCATT GGTACC GCG GCG GGATCC ACACACACGGTCACCGTTAACCTC | |
| *rtxB28* | F | GGACTGGGCATTTTTTTTCATTAT GGATCC GCG GCG GGTACC CGTAGTGCTCCAGTTGCCTGCAGC | |
| *rtxB28* | R | GCTGCAGGCAACTGGAGCACTACG GGTACC GCG GCG GGATCC ATAATGAAAAAAAATGCCCAGTCC | |
| *hlyB28* | F | CCTTGCTGGCGAATCATCAGTTGATGGTGGTGTAGATA GTGTAGGCTGGAGCTGCTTCG | |
| *hlyB28* | R | GGGTTCAGTCAGACCACGGTTATATTGCAGTTGTCCGT CATATGAATATCCTCCTTAG | |
| *hlyD28* | F | CGGGTGGTGTTGCAGGAGAAGAATAAGGGGTCTGCGGC GTGTAGGCTGGAGCTGCTTCG | |
| *hlyD28* | R | GCTGGTCAGGCCGGTAATTTTTGCTACATCGCTAATGT CATATGAATATCCTCCTTAG | |
| Primers for mutant detection | | | |
| OI-28 | F | GTTGTTGGTGATCGTTGGCT | |
| OI-28 | R | GCGCAGTAAGGTCAATTCGT | |
| Primers for complement construction | | | |
| *tolC28* | F | GGTTCATATGATGGCGCGATTCCAGTTTAAAAAAC | |
| *tolC28* | R | GGTTCTCGAGTTTCAAGTCGGGTAAGTCGTTGTAT | |
| *rtxA28* | F | CGCCATATGATGAGCCTGATTATTGATG TTA | |
| *rtxA28* | R | CCCAAGCTTCTAATTGTCGCTGTTGCCTGCC | |
| *rtxB28* | F | GGTTCATATGGTGCCAGCTTCCGCAGTAGGT | |
| *rtxB28* | R | GGTTCTCGAGCACACCACCGCCGTCACCAACG | |
| *hlyB28* | F | GGTTCATATGATGAAAAAAAATGCCCAGTCC | |
| *hlyB28* | R | GGTTCTCGAGTTCCAGTCACCGACGAGGCT | |
| *hlyD28* | F | GGTTCATATGATGAACGGACAACTGCAATAT | |
| *hlyD28* | R | GGTTCTCGAGTCATCGTCGTTT GTTAAAGGG | |
| Primers for protein purification | | | |
| *rstA* | F | CATGCCATGG GTGAATGTTATGAACACTATCGTA | |
| *rstA* | R | CGCGGATCC TTATTCCCATGCATGAGGCGCAAA | |
| Primers for EMSA | | | |
| *rpos* | | F | GTTATCGCAGGGAGCCACA |
| *rpos* | | R | TTTTACCACCAGACGCAAGTTA |
| *asr* | | F | AGACTGCGACCACACCTGCTCC |
| *asr* | | R | GCGGGTTGTGCAGCAGTTTTG |
| *tolC28* | | F | GATC AAGTTG TAATTCAAATTACC TTGAC |
| *tolC28* | | R | GTCAA GGT AATTTGAATTAA CAACTT |
| Primers for RstA-3×FLAG | | | |
| RstA-3F | | F | CGGGAATTCATGATGCGGAGTGTAACCATGAAA |
| RstA-3R | | R | CGCGGATCCTTACTATTTATCGTCGTCATCTTTGTAGTCGATATCATGATCTTTATAATCACCGTCATGGTCTTTGTAGTCATGCACGTCGGCAGCGAAGAC |
| Primers for ChIP-qPCR | | | |
| *tolC28* | | F | GCTGACGATTTGCTGTTGTT |
| *tolC28* | | R | CCGTTTATCAGCGTTGATGG |
| *rpoS* | | F | GTTATCGCAGGGAGCCACA |
| *rpoS* | | R | TTTTACCACCAGACGCAAGTTA |
| Primers for qRT-PCR | | | |
| *tolC28* | | F | ACTTACGCCAGTCAGCACAGTACG |
| *tolC28* | | R | AGCACATCCAGTTTTGCCTGTTCG |
| *rtxA28* | | F | CCATCAACACCATCGCTCAGGACA |
| *rtxA28* | | R | TTGACAGCGTAATTACCCTCCGCC |
| *rtxB28* | | F | CGCAGGCCACCGGTTTTAACAC |
| *rtxB28* | | R | CGCAGGCCACCGGTTTTAACAC |
| *hlyB28* | | F | AGTTAGGGATGGGGTTGCGGTTG |
| *hlyB28* | | R | CCCTTCGTCACCGCTGAACTGAA |
| *hlyD28* | | F | TGGACGGGAAGACTCTCTCACCAG |
| *hlyD28* | | R | GCCGCCGTAGATGGAGTAATCGTA |
| *rpoA* | | F | GCGCTCATCTTCTTCCGAAT |
| *rpoA* | | R | CGCGGTCGTGGTTATGTG |
